# Occurrence dataset of waterbirds in the Tiaozini Wetland, a World Nature Heritage, China

**DOI:** 10.3897/BDJ.10.e90724

**Published:** 2022-10-03

**Authors:** Wei Hu, Taiyu Chen, Zheping Xu, Dawei Wu, Changhu Lu

**Affiliations:** 1 College of Biology and the Environment, Nanjing Forestry University, Nanjing, China College of Biology and the Environment, Nanjing Forestry University Nanjing China; 2 National Science Library, Chinese Academy of Sciences, Beijing, China National Science Library, Chinese Academy of Sciences Beijing China

**Keywords:** Tiaozini Wetland, World Nature Heritage, waterbirds, endangered species, dataset

## Abstract

**Background:**

Tiaozini, the core area of the Yellow (Bohai) Sea Migratory Bird Habitat in Dongtai, Jiangsu Province and a World Heritage Site, has provided an ideal habitat for migratory birds. As an important hub on the East Asian–Australasian Flyway (EAAF), Tiaozini Wetland provides pivotal stopover and wintering sites for tens of thousands of migratory waterbirds, including some global critically endangered species, such as Spoon-billed Sandpiper (*Calidrispygmaea*) and Spotted Greenshank (*Tringaguttifer*). Although many researchers have conducted a lot of studies on waterbirds in Tiaozini Wetland, there is still a lack of a dataset on waterbird species composition and individual quantity in Tiaozini Wetland throughout the year. Here, we conducted a one-year waterbird survey in the Tiaozini Wetland during 2020-2021 and provided an occurrence dataset with detailed species and geographic information.

**New information:**

This occurrence dataset is the first public record of species and number of waterbirds in Tiaozini Wetland for a whole year, which includes the taxonomic information, location information, number, investigation date and endangered level for each species. All data have been published on GBIF.

## Introduction

The East Asian–Australasian Flyway (EAAF) is one of the nine major migratory bird migration routes in the world, with a total length of about 12,000 kilometres, spanning 22 countries and nearly 50 million waterbirds migrating on this route, which is the most crowded and also the most threatened migratory route for migratory birds (Chen 2015). As the centre of the EAAF, China's coastal wetlands provide habitats for more than 200 migratory waterbirds species and over 70% of the globally-threatened waterbirds species on the EAAF depend on China's Yellow and Bohai Seas intertidal wetlands (Xia et al. 2017). However, in the past 50 years, due to the substantial increase in land demand caused by population growth and economic development, about 40% of China's coastal wetlands have been degraded or disappeared (Xia et al. 2017). Coastal wetlands reclamation, environmental pollution and biological invasion have caused the loss and fragmentation of waterbirds' habitats, which have seriously threatened the survival of waterbirds on EAAF (Peng et al. 2017).

The Tiaozini Wetland is located in Dongtai, Jiangsu Province, China, which is an important stopover and wintering habitat for migratory waterbirds on the EAAF (Tong et al. 2012, Gao et al. 2021). Amongst the waterbirds inhabiting in the Tiaozini Wetland, 21 species accounted for more than 1% of the total species on the EAAF (Bai et al. 2015). At the same time, about 40% of the world's Spoon-billed Sandpiper population moult in the Tiaozini Wetland and the number of Spotted Greenshank recorded in the Tiaozini Wetland is twice the estimated world population of this species in 2016 (Bai et al. 2015, Chang et al. 2019). Therefore, the Tiaozini Wetland is not only a pivotal area for endangered species protection, but also a hotspot for waterbirds observation and waterbirds research. Considering the importance of the Tiaozini Wetland for migrating waterbirds on the EAAF, on 5 July 2019, the first phase of the China Yellow and Bohai Seas Migratory Bird Habitat, which is located in the Tiaozini Wetland, was listed as a World Natural Heritage Site by UNESCO and it was also the first intertidal Wetland Heritage Site in China and the second in the world (IUCN 2019, Wang et al. 2021). Therefore, many bird researchers have carried out various studies in the Tiaozini Wetland. Clark et al. (2016) first accurately estimated the global population of Spoon-billed Sandpiper by surveys in Tiaozini, Yangkou and Dongling in 2014 (Clark et al. 2016); Gao et al. (2021) investigated the diversity index of waterbird communities in the Tiaozini and Rudong Wetlands during the overwintering period from 2017 to 2020 (Gao et al. 2021); Sun et al. (2021) studied the habitat selection of waterbirds in coastal wetlands for the impact of reclamation on migratory waterbirds during the overwintering period from 2018 to 2019 in Jiangsu Province (Sun et al. 2021). In addition, many researchers used published data to study habitat suitability, network structure and function in the Tiaozini Wetland (Duan et al. 2020, Wang et al. 2022). However, most studies only recorded and published waterbirds data on part of the species and some months. The annual list of waterbirds in the Tiaozini Wetland was not published, so we investigated the species composition and waterbirds quantities in the Tiaozini Wetland throughout the year and provided occurrence data in order to support waterbird diversity research and endangered species protection in the future.

## Sampling methods

### Sampling description

**Figure 1.**
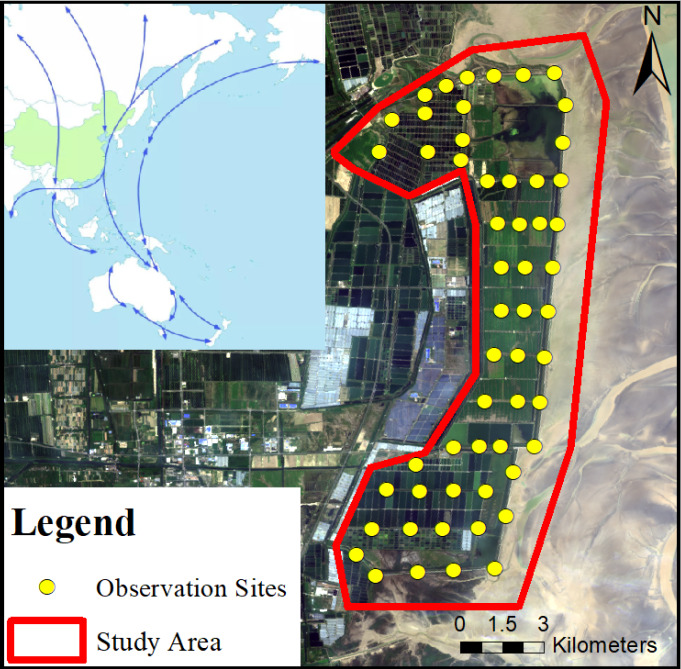
Location of observation sites and study area.

We used the sampling points method to investigate species composition and individual quantity of waterbirds in Tiaozini Wetland. After the pre-survey fieldwork, we set up 55 observation points with an interval of about 1 km (Fig. 1). The waterfowl survey was conducted on a monthly basis during good weather conditions, within a 2 h time window before and after high tide according to the local tidal table. The time spent at each observation site was approximately 4 minutes and the observation area was a circle area with a 500 m radius centred on the observation site. The species and quantity of waterbirds in the field of view were counted using Shuntu 8 x 42 binoculars and Nikon 10 x 60 monoculars and waterbirds were photographed and recorded using a Canon 6D2 camera with external 150-600 mm lens. To save time and avoid duplicate counts, three groups of experienced researchers (two in each group) conducted surveys simultaneously in different areas of the Tiaozini Wetland. Only waterbirds staying or flying into the observation area were recorded, while waterbirds flying out of the observation area were not recorded. For bird groups that were easy to identify and small in number, the species and quantity of birds were recorded directly using the count method; for bird groups that were difficult to identify and large in number, the quantity of waterbird clusters and the proportion of each species were estimated using the photo-taking method, followed by identification. Classification of waterbirds was undertaken according to *A Checklist on the Classification and Distribution of the Birds of China (Third Edition)* (Zheng 2017). Collation and summary of waterbirds data were carried out after the daylight fieldwork by using Microsoft Office Excel 2021 and the data setwas organised according to the Darwin Core format and uploaded to GBIF after the one-year survey (Hu et al. 2022).

## Geographic coverage

### Description

We downloaded the Landsat8 satellite image on 18 September 2020 and drew the investigation scope by using ArcGIS 10.7 software. Our survey covered almost all areas of Tiaozini Wetland, such as estuary, intertidal, aquaculture pond and farmland.

### Coordinates

32.71N and 32.89N Latitude; 120.89E and 120.97 E Longitude.

## Taxonomic coverage

### Description

A total of 51231 waterbirds were recorded in this occurrence dataset, belonging to 104 species, 16 families and nine orders (Table 1). Many species were included in the China Species Red List and the IUCN Red List (Wang and Xie 2009, IUCN 2021). In the China Species Red List, *Calidrispygmaea* (Linnaeus, 1758), *Tringaguttifer* (Nordmann, 1835), *Saundersilarussaundersi* (Swinhoe, 1871), *Ciconiaboyciana* (Swinhoe, 1873), *Plataleaminor* (Temminck & Schlegel, 1849) and *Pelecanuscrispus* (Bruch, 1832) were ranked as National First-class Protected Animals; *Anseralbifrons* (Scopoli, 1769), *Cygnuscolumbianus* (Ord, 1815), *Aixgalericulata* (Linnaeus, 1758), *Mergellusalbellus* (Linnaeus, 1758), *Podicepsnigricollis* (Brehm, 1831), *Limnodromussemipalmatus* (Blyth, 1848), *Numeniusminutus* (Gould, 1841), *Numeniusarquata* (Linnaeus, 1758), *Numeniusmadagascariensis* (Linnaeus, 1766), *Arenariainterpres* (Linnaeus, 1758), *Calidristenuirostris* (Horsfield, 1821), *Calidrisfalcinellus* (Pontoppidan, 1763) and Platalealeucorodia (Linnaeus, 1758) were ranked as National Second-class Protected Animals. In the IUCN Red List, *Calidrispygmaea* (Linnaeus, 1758) was ranked as Critically Endangered (CR); *Numeniusmadagascariensis* (Linnaeus, 1766), *Tringaguttifer* (Nordmann, 1835), *Calidristenuirostris* (Horsfield, 1821), *Ciconiaboyciana* (Swinhoe, 1873) and *Plataleaminor* (Temminck & Schlegel, 1849) were ranked as Endangered (EN); *Aythyaferina* (Linnaeus, 1758) and *Saundersilarussaundersi* (Swinhoe, 1871) were ranked as Vulnerable (VU); *Marecafalcata* (Georgi, 1775), *Aythyanyroca* (Güldenstädt, 1770), *Haematopusostralegus* (Linnaeus, 1758), *Vanellusvanellus* (Linnaeus, 1758), *Limnodromussemipalmatus* (Blyth, 1848), *Limosalimosa* (Linnaeus, 1758), *Limosalapponica* (Linnaeus, 1758), *Numeniusarquata* (Linnaeus, 1758), *Tringabrevipes* (Vieillot, 1816), *Calidriscanutus* (Linnaeus, 1758), *Calidrisruficollis* (Pallas, 1776), *Calidrisferruginea* (Pontoppidan, 1763) and *Pelecanuscrispus* (Bruch, 1832) were ranked as Near Threatened (NT). The reason for the rank differences of some waterbird species between the China Species Red List and IUCN Red List is due to the large difference between the distribution quantity of these species in the world and China.

## Temporal coverage

### Notes

This survey was conducted monthly from July 2020 to June 2021. The specific dates were: 2020/07/16; 2020/08/15; 2020/09/26; 2020/10/30; 2020/11/17; 2020/12/07; 2021/01/17; 2021/02/21; 2020/03/18; 2021/04/15; 2021/05/21; 2021/06/16.

## Usage licence

### Usage licence

Creative Commons Public Domain Waiver (CC-Zero)

### IP rights notes

Creative Commons Attribution Non Commercial (CC-BY-NC) 4.0 License

## Data resources

### Data package title

Occurrence dataset of waterbirds in the Tiaozini Wetland, the World Nature Heritage, China

### Resource link

https://www.gbif.org/dataset/4c3b430e-1b2b-4668-9f07-6c8d7cf60e2e#dataDescription

### Alternative identifiers

https://doi.org/10.15468/npfwev

### Number of data sets

1

### Data set 1.

#### Data set name

Occurrence dataset of waterbirds in the Tiaozini Wetland, the World Nature Heritage, China

#### Data format

Darwin Core Archive format

#### Download URL

https://www.gbif.org/occurrence/download?dataset_key=4c3b430e-1b2b-4668-9f07-6c8d7cf60e2e

#### Description

Our occurrence data contains 30 column labels and all data are georeferenced. Due to the limitations of bird observation, the coordinates of all species are replaced by the coordinates of the observation site.

**Data set 1.** 

Column label	Column description
occurrenceID	An identifier for the bird occurrence.
basisOfRecord	The specific nature of the data record.
licence	A legal document giving official permission to do something with the resource.
ownerInstitutionCode	The name (or acronym) in use by the institution having ownership of the object(s) or information referred to in the record.
recordedBy	A list (concatenated and separated) of names of people, groups or organisations responsible for recording the original Occurrence. The primary collector or observer, especially one who applies a personal identifier (recordNumber), should be listed first.
individualCount	The number of individuals present at the time of the Occurrence.
occurrenceStatus	A statement about the presence or absence of a Taxon at a Location.
eventDate	The date when the event was recorded.
year	The four-digit year in which the Event occurred, according to the Common Era Calendar.
month	The integer month in which the Event occurred.
day	The integer day of the month on which the Event occurred.
countryCode	The standard code for the country in which the Location occurs.
stateProvince	The name of the next smaller administrative region than country (state, province, canton, department, region etc.) in which the Location occurs.
county	The full, unabbreviated name of the next smaller administrative region than stateProvince (county, shire, department etc.) in which the Location occurs.
locality	The specific description of the place.
decimalLatitude	The geographic latitude of the geographic centre of a Location.
decimalLongitude	The geographic longitude of the geographic centre of a Location.
scientificName	The full scientific name, with authorship and date information, if known. When forming part of an Identification, this should be the name in lowest level taxonomic rank that can be determined. This term should not contain identification qualifications, which should instead be supplied in the IdentificationQualifier term.
kingdom	The full scientific name of the kingdom in which the taxon is classified.
phylum	The full scientific name of the phylum in which the taxon is classified.
class	The full scientific name of the class in which the taxon is classified.
order	The full scientific name of the order in which the taxon is classified.
family	The full scientific name of the family in which the taxon is classified.
genus	The full scientific name of the genus in which the taxon is classified.
genericName	The genus part of the scientificName without authorship.
specificEpithet	The name of the first or species epithet of the scientificName.
taxonRank	The taxonomic rank of the most specific name in the scientificName as it appears in the original record.
vernacularName	A common or vernacular name.
taxonomicStatus	The status of the use of the scientificName as a label for a taxon. Requires taxonomic opinion to define the scope of a taxon. Rules of priority then are used to define the taxonomic status of the nomenclature contained in that scope, combined with the experts opinion. It must be linked to a specific taxonomic reference that defines the concept.
taxonRemarks	Comments or notes about the taxon or name.

## Figures and Tables

**Table 1.** Waterbirds list in the Tiaozini Wetland.

Rank	Order	Family	Scientific name	Number of observations
1	Anseriformes	Anatidae	*Anserfabalis*	70
2	Anseriformes	Anatidae	*Anseralbifrons*	1
3	Anseriformes	Anatidae	*Cygnuscolumbianus*	5
4	Anseriformes	Anatidae	*Tadornatadorna*	358
5	Anseriformes	Anatidae	*Tadornaferruginea*	38
6	Anseriformes	Anatidae	*Aixgalericulata*	2
7	Anseriformes	Anatidae	*Marecastrepera*	98
8	Anseriformes	Anatidae	*Marecafalcata*	354
9	Anseriformes	Anatidae	*Marecapenelope*	47
10	Anseriformes	Anatidae	*Anasplatyrhynchos*	426
11	Anseriformes	Anatidae	*Anaszonorhyncha*	2097
12	Anseriformes	Anatidae	*Anasacuta*	23
13	Anseriformes	Anatidae	*Anascrecca*	1475
14	Anseriformes	Anatidae	*Spatulaclypeata*	135
15	Anseriformes	Anatidae	*Spatulaquerquedula*	25
16	Anseriformes	Anatidae	*Aythyaferina*	861
17	Anseriformes	Anatidae	*Aythyanyroca*	4
18	Anseriformes	Anatidae	*Aythyafuligula*	502
19	Anseriformes	Anatidae	*Aythyamarila*	16
20	Anseriformes	Anatidae	*Mergellusalbellus*	2
21	Anseriformes	Anatidae	*Mergusmerganser*	30
22	Podicipediformes	Podicipedidae	*Tachybaptusruficollis*	285
23	Podicipediformes	Podicipedidae	*Podicepscristatus*	36
24	Podicipediformes	Podicipedidae	*Podicepsnigricollis*	7
25	Phoenicopteriformes	Phoenicopteridae	*Phoenicopterusroseus*	14
26	Gruiformes	Rallidae	*Gallinulachloropus*	152
27	Gruiformes	Rallidae	*Fulicaatra*	10188
28	Charadriiformes	Haematopodidae	*Haematopusostralegus*	263
29	Charadriiformes	Recurvirostridae	*Himantopushimantopus*	374
30	Charadriiformes	Recurvirostridae	*Recurvirostraavosetta*	4175
31	Charadriiformes	Charadriidae	*Vanellusvanellus*	45
32	Charadriiformes	Charadriidae	*Vanelluscinereus*	73
33	Charadriiformes	Charadriidae	*Pluvialisfulva*	109
34	Charadriiformes	Charadriidae	*Pluvialissquatarola*	814
35	Charadriiformes	Charadriidae	*Charadriusdubius*	98
36	Charadriiformes	Charadriidae	*Charadriusalexandrinus*	1438
37	Charadriiformes	Charadriidae	*Charadriusmongolus*	288
38	Charadriiformes	Charadriidae	*Charadriusleschenaultii*	795
39	Charadriiformes	Scolopacidae	*Scolopaxrusticola*	2
40	Charadriiformes	Scolopacidae	*Gallinagogallinago*	13
41	Charadriiformes	Scolopacidae	*Limnodromusscolopaceus*	2
42	Charadriiformes	Scolopacidae	*Limnodromussemipalmatus*	38
43	Charadriiformes	Scolopacidae	*Limosalimosa*	1360
44	Charadriiformes	Scolopacidae	*Limosalapponica*	1214
45	Charadriiformes	Scolopacidae	*Numeniusminutus*	3
46	Charadriiformes	Scolopacidae	*Numeniusphaeopus*	84
47	Charadriiformes	Scolopacidae	*Numeniusarquata*	1563
48	Charadriiformes	Scolopacidae	*Numeniusmadagascariensis*	52
49	Charadriiformes	Scolopacidae	*Tringaerythropus*	149
50	Charadriiformes	Scolopacidae	*Tringatotanus*	378
51	Charadriiformes	Scolopacidae	*Tringastagnatilis*	158
52	Charadriiformes	Scolopacidae	*Tringanebularia*	546
53	Charadriiformes	Scolopacidae	*Tringaguttifer*	65
54	Charadriiformes	Scolopacidae	*Tringaochropus*	14
55	Charadriiformes	Scolopacidae	*Tringaglareola*	30
56	Charadriiformes	Scolopacidae	*Tringabrevipes*	22
57	Charadriiformes	Scolopacidae	*Xenuscinereus*	170
58	Charadriiformes	Scolopacidae	*Actitishypoleucos*	22
59	Charadriiformes	Scolopacidae	*Arenariainterpres*	59
60	Charadriiformes	Scolopacidae	*Calidristenuirostris*	148
61	Charadriiformes	Scolopacidae	*Calidriscanutus*	33
62	Charadriiformes	Scolopacidae	*Calidrisalba*	1581
63	Charadriiformes	Scolopacidae	*Calidrisruficollis*	4301
64	Charadriiformes	Scolopacidae	*Calidrispygmaea*	31
65	Charadriiformes	Scolopacidae	*Calidrisminuta*	3
66	Charadriiformes	Scolopacidae	*Calidristemminckii*	20
67	Charadriiformes	Scolopacidae	*Calidrissubminuta*	69
68	Charadriiformes	Scolopacidae	*Calidrisacuminata*	754
69	Charadriiformes	Scolopacidae	*Calidrisfalcinellus*	144
70	Charadriiformes	Scolopacidae	*Calidrispugnax*	1
71	Charadriiformes	Scolopacidae	*Calidrisferruginea*	381
72	Charadriiformes	Scolopacidae	*Calidrisalpina*	5281
73	Charadriiformes	Scolopacidae	*Phalaropuslobatus*	5
74	Charadriiformes	Glareolidae	*Glareolamaldivarum*	281
75	Charadriiformes	Laridae	*Chroicocephalusridibundus*	821
76	Charadriiformes	Laridae	*Saundersilarussaundersi*	983
77	Charadriiformes	Laridae	*Laruscrassirostris*	179
78	Charadriiformes	Laridae	*Larussmithsonianus*	294
79	Charadriiformes	Laridae	*Laruscachinnans*	2
80	Charadriiformes	Laridae	*Larusschistisagus*	41
81	Charadriiformes	Laridae	*Gelochelidonnilotica*	160
82	Charadriiformes	Laridae	*Hydroprognecaspia*	583
83	Charadriiformes	Laridae	*Sternulaalbifrons*	231
84	Charadriiformes	Laridae	*Sternahirundo*	341
85	Charadriiformes	Laridae	*Chlidoniashybrida*	63
86	Charadriiformes	Laridae	*Chlidoniasleucopterus*	34
87	Gaviiformes	Gaviidae	*Gaviastellata*	1
88	Ciconiiformes	Ciconiidae	*Ciconiaboyciana*	23
89	Suliformes	Phalacrocoracidae	*Phalacrocoraxcarbo*	341
90	Pelecaniformes	Threskiornithidae	*Platalealeucorodia*	479
91	Pelecaniformes	Threskiornithidae	*Plataleaminor*	227
92	Pelecaniformes	Ardeidae	*Botaurusstellaris*	1
93	Pelecaniformes	Ardeidae	*Ixobrychussinensis*	2
94	Pelecaniformes	Ardeidae	*Ixobrychuseurhythmus*	1
95	Pelecaniformes	Ardeidae	*Nycticoraxnycticorax*	129
96	Pelecaniformes	Ardeidae	*Butoridesstriata*	3
97	Pelecaniformes	Ardeidae	*Ardeolabacchus*	15
98	Pelecaniformes	Ardeidae	*Bubulcusibis*	91
99	Pelecaniformes	Ardeidae	*Ardeacinerea*	439
100	Pelecaniformes	Ardeidae	*Ardeapurpurea*	2
101	Pelecaniformes	Ardeidae	*Ardeaalba*	264
102	Pelecaniformes	Ardeidae	*Ardeaintermedia*	19
103	Pelecaniformes	Ardeidae	*Egrettagarzetta*	721
104	Pelecaniformes	Pelecanidae	*Pelecanuscrispus*	41
